# Functional Properties of a Cysteine Proteinase from Pineapple Fruit with Improved Resistance to Fungal Pathogens in *Arabidopsis thaliana*

**DOI:** 10.3390/molecules19022374

**Published:** 2014-02-21

**Authors:** Wei Wang, Lu Zhang, Ning Guo, Xiumei Zhang, Chen Zhang, Guangming Sun, Jianghui Xie

**Affiliations:** 1 Anhui Key Laboratory of Plant Genetic & Breeding, School of Life Science, Anhui Agricultural University, 130 Changjiang West Road, Hefei 230036, China; E-Mails: wangweisys@ahau.edu.cn (W.W.); guoning@ahau.edu.cn (N.G.); zhangchen@ahau.edu.cn (C.Z.); 2 Key Laboratory of Tropical Fruit Biology, Ministry of Agriculture, Institute of China Southern Subtropical Crop Research, Chinese Academy of Tropical Agricultural Sciences (CATAS), Zhanjiang 524091, Guangzhou, China; E-Mails: xiumeizhang1980@163.com (X.Z.); guangmingsun52@163.com (G.S.); 3 State Key Laboratory of Biocontrol, School of Life Sciences, SunYat-sen University, 510006 Guangzhou, China; E-Mail: luzhangtest@163.com

**Keywords:** *Ananas comosus*, cysteine proteinase, gene expression, proteolytic properties, resistance

## Abstract

In plant cells, many cysteine proteinases (CPs) are synthesized as precursors in the endoplasmic reticulum, and then are subject to post-translational modifications to form the active mature proteinases. They participate in various cellular and physiological functions. Here, AcCP2, a CP from pineapple fruit (*Ananas comosus* L.) belonging to the C1A subfamily is analyzed based on the molecular modeling and homology alignment. Transcripts of *AcCP2* can be detected in the different parts of fruits (particularly outer sarcocarps), and gradually increased during fruit development until maturity. To analyze the substrate specificity of AcCP2, the recombinant protein was overexpressed and purified from *Pichia pastoris*. The precursor of purified AcCP2 can be processed to a 25 kDa active form after acid treatment (pH 4.3). Its optimum proteolytic activity to Bz-Phe-Val-Arg-NH-Mec is at neutral pH. In addition, the overexpression of *AcCP2* gene in *Arabidopsis thaliana* can improve the resistance to fungal pathogen of *Botrytis cinerea*. These data indicate that AcCP2 is a multifunctional proteinase, and its expression could cause fruit developmental characteristics of pineapple and resistance responses in transgenic *Arabidopsis* plants.

## 1. Introduction

Cysteine proteinases (EC 3.4.22, CPs), known as thiol proteinases, are widely distributed among living organisms. CPs in plants mainly participate in key cellular and physiological functions such as development and germination of seeds, leaf and flower senescence, fruit ripening and resistance responses to biotic and abiotic stresses, *etc.* [1,2,3,4,5,6,7]. To date, more than 101 families of CPs were registered in the MEROPS database (last entry from October, 2013) [8]. They are grouped into at least nine clans according to the different folds in the tertiary structure. The best known CP is the papain family (clan CA, family C1). Papain proteinases are synthesized as an inactive precursor with a signal peptide, an N-terminal inhibitory pro-region as well as a mature catalytic domain [9]. Mature papain-like proteinases share similar catalytic residues in the order of Cys....His....Asn/Asp and the three-dimensional structure [10,11]. They are characterized by the presence of multiple disulfide bridges, and mainly accumulate in the vacuole, apoplast or specific vesicles [12,13]. There are also differences in the optimum pH of papain-like CPs owing to the structure and amino acid components [14].

An important source of plant proteinases used in the traditional medicine and industry is bromelain, which is actually a mixture of different CPs with similar amino acid sequences [15,16,17]. These enzymes display different proteolytic activities and molecular masses ranging from 20 to 38 kDa [18]. However, it is not still clear how these different proteinases within crude bromelain contribute to their functional activity *in vivo*. Studies to test the potential efficacy of bromelain indicate that at least five distinct AcCPs [stem bromelain (EC 3.4.22.32), acidic stem bromelain, fruit bromelain (EC 3.4.22.33), ananain (EC 3.4.22.31) and comosain] belonging to the papain family could take part in different physiological processes [11,16,17,19]. Several synthetic peptide substrates (such as Z-Arg-Arg-NH-Mec and Bz-Phe-Val-Arg-NH-Mec) had been used to characterize the proteolytic activity of purified bromelain enzymes [19,20,21,22]. Until now, more than six major full-length genes of CPs from bromelain complex have been identified in the MEROPS database, which are comprised of stem bromelain and fruit bromelain. By contrast, fruit CP (O23791) and stem CP (ADY68475) share 87% cDNA sequence identity [22,23]. In stem bromelain, two homology genes named comosain and ananain have been also isolated, but there are no reports of AcCP isoforms of fruit bromelain. Although the physicochemical properties of fruit and stem bromelains are very well characterized [18,20,22,24], their *in vivo* roles are not yet completely understood, especially in the development and ripening of pineapple fruits. Therefore, the current research mainly focuses on functional properties of a CP isolated from pineapple fruit library. The expression patterns of *AcCP2* in tissues and sugar accumulation are also examined. The results reveal that transcription levels of *AcCP2* might be correlated to the fruit softness and maturity. Currently, although many approaches have been used to improve the purity and activity of bromelain enzyme preparations, they require expensive materials and complicated processes [15]. Because of the benefits of yeast expression system [25], *AcCP2* was expressed in *Pichia pastoris* to study the detailed enzymatic properties and possible biotechnological application using synthetic substrates *in vitro*. In addition, transgenic *Arabidopsis* plants overexpressing the *AcCP2* gene demonstrate the improved resistance to fungal pathogens.

## 2. Results and Discussion

### 2.1. Molecular Cloning of the AcCP2 Gene

**Figure 1:**
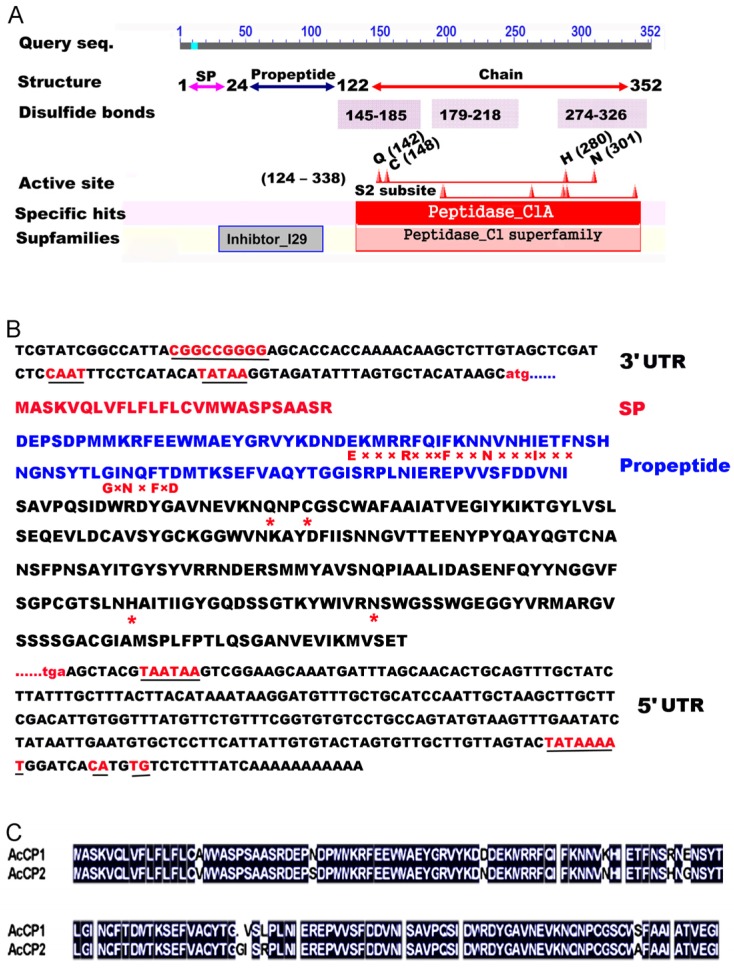
Representation of AcCP2 domains and deduced amino acid sequences. (**A**) Schematic demonstration of AcCP2 structure characteristics; (**B**) Deduced amino acid sequences of AcCP2. The letters underlined in nucleotide sequences indicate the CAAT box, TATA box and GC box in the upstream of start codon. CA-GU sequences are regarded as an alternative site combining PolyA synthase in higher plants. The consensus polyadenylation signal (AATAAA) is found in 3' UTR. SP, GxNxFxD and ERFNIN motifs are also noted in the propeptide. Conserved active sites (Gln, Cys, His and Asn) are labeled in asterisks (*); and (**C**) Alignment of amino acid sequences between AcCP1 (O23791) and AcCP2 (AEH26024)*.*

For isolating the genes related to fruit development and ripening, cDNA libraries of pineapple fruits were constructed into pGEM-T easy vector using mRNAs of different developmental stages. Of these sequences, 12 fragments showed significant identity to CPs of *A. comosus*. The RACE method was adopted to amplify the full-length genes. Interestingly, a full-length cDNA including an open reading frame of 1 059 bp and two untranslated regions (5'-102 bp and 3'-253 bp) was obtained and the gene was *AcCP2*, the homologous gene of fruit bromelain. The sequence was deposited in GenBank (ID: JF831511). AcCP2 was predicted to contain a signal peptide (SP) preceding the N-terminal sequence (1–24 amino acids), suggesting an extracellular localization. The prosequence residues 24–122 precede the mature enzyme residues 122–352 (Figure 1A). As could be expected, the predicted AcCP2 protein contains several of characteristic elements of papain structure and active sites (Figure 1A,B), which are essential for catalytic activity and maintaining the tertiary structure [26]. Location and functions of the *cis*-elements were predicated by PlantCARE database search program. Two highly conserved motifs found in most papain-like CP propeptides, GxNxFxD and ERFNIN seem to be essential for the correct processing of proteinase precursors (Figure 1B) [27]. The sequence of AcCP2 is homologous to CPs from Bromeliaceae, sharing 88% identity with stem bromelain (ADY68475), and 87% identity with macrodontain I (P83443). Although the highest homology is found between fruit bromelain (O23791) and AcCP2, there are some differences in functional domains and length (Figure 1C). In addition, AcCP2 has the lower similarity to other proteinases, 40%, 44% and 47% identity with papain (P00784), *A. thaliana* (BAC43602) and *Oryza sativa* (CAH66275), respectively.

### 2.2. Structure Characteristics of the AcCP2 Protein

N- and C- terminal domains of CP from different species display conserved characteristics (see multiple sequence alignment in Figure S1A). In overall structure predicted by the template of papain, the mature protein fold is composed of two domains with the catalytic site lying between them. The left (L) domain (N-terminal domain) consists of some helices and active sites of Gln20 and Cys26. The right (R) domain contains His158 and Asn179 residues (Figure S1B). Three disulfide bonds (23–57, 63–96 and 152–204) could play a key role in maintaining the three dimensional structure [13]. Analysis of the phylogenetic tree also clearly shows that AcCP2 belongs to CA:C1 family and is more closely related to each other from pineapple stems and fruits than to other members of papain family (Figure S1C).

### 2.3. Expression Patterns of AcCP2 During Pineapple-Fruit Development

**Figure 2:**
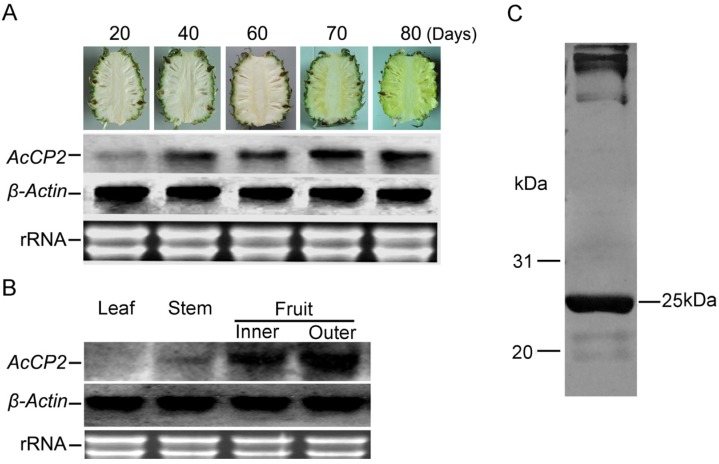
Temporal and spatial transcript levels of *AcCP2* are analyzed by Northern blotting with specific probe primers. To ensure equal sample abundance on gels, rRNAs are quantified to monitor loading equivalence. *β-Actin* gene is used as an internal control. The data is used for analysis from at least three independent experiments ± SE (*n* = 3). (**A**) The characteristics of *AcCP2* transcription during fruit development and ripening; (**B**) Expression levels of *AcCP2* in different tissues of pineapple; and (**C**) AcCP protein detection in pineapple fruits using purified antibodies.

Although the transcripts of *AcCP2* can be detected during development and ripening of pineapple fruits using Northern blot, continuous *AcCP2* mRNA differences are found in contrast to expression levels of β-actin (Figure 2A). As shown in Figure 2B, *AcCP2* is strongly expressed in mature fruits, especially in the outer sarcocarps. However, RNAs from leaf and stem show significantly lower hybridization signals. From 20 to 70 days after anthesis, the transcript levels of *AcCP2* mRNA are gradually increased to 10-fold in fruit samples. No significant differences are observed between 70 and 80 days. The results suggest that expression of *AcCP2* could be involved in the growth and development of pineapple fruits. Fruit bromelain is isolated and analyzed using western blot. A distinct ≈ 25 kDa band can be detected using purified antibodies (Figure 2C).

### 2.4. Expression of His-AcCP2 Recombinant Protein

**Figure 3:**
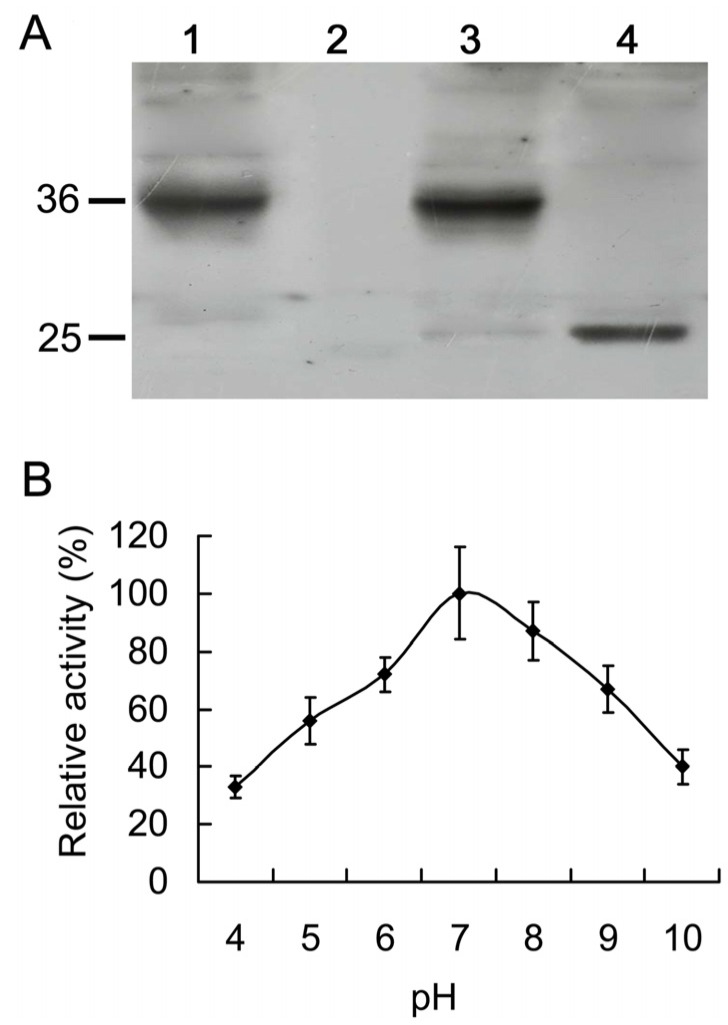
Protein purification and proteolytic activity of AcCP2 expressed in yeast. (**A**) Immuno-detection of recombinant His-AcCP2 protein. Lane 1: AcCP2 crude proteins extracted from yeast containing the pPIC9K-His-*AcCP2* vector; Lane 2: crude proteins extracted from yeast containing the pPIC9K empty vector; Lane 3: protein purification by Ni-NTA beads from yeast containing the pPIC9K-His-*AcCP2* vector; Lane 4: the purified protein incubated at pH 4.3; and (**B**) Activity was measured on 1% Bz-Phe-Val-Arg-NH-Mec solution containing 15 mM AcCP2 proteinase under different pH. Data points represent the mean values of three replicates ± SE (*n* = 3).

The pPIC9K-*AcCP2* plasmids were expressed in *P. pastoris* for exploring the enzymatic properties and possible biotechnological applications of AcCP2. Western blot shows a ≈36 kDa protein which corresponds to the protein precursor plus the His6x tag. Regardless of the success of expression of the protein in yeast, the propeptide has to be removed from the protein to be active, even if it is a natural product [28]. AcCP2 protein was purified using Ni-NTA beads, and then treated in acetate buffer at pH 4.3 to induce autoactivation. An approximately 25 kDa single band was demonstrated on the nitrocellulose membrane, whereas unprocessed protein is ≈36 kDa (Figure 3A).

### 2.5. Catalytic Characteristics of His-AcCP2 Recombinant Protein Using Synthetic Substrates

To understand the nature of substrates for AcCP2 protein expressed in yeast, catalytic and specific activities were analyzed using Bz-Phe-Val-Arg-NH-Mec and Z-Arg-Arg-NH-Mec. The activities of purified AcCP2 protein and commercial fruit bromelain using equivalent amounts were determined against two synthetic substrates. Two samples also have specific activities against the Bz-Phe-Val-Arg-NH-Mec substrate, whereas they show minimal activities against Z-Arg-Arg-NH-Mec substrate (Table 1). The purified AcCP2 shows a broad range of activity from pH 4.0 to 10.0, and has the optimum pH ≈ 7.0 using Bz-Phe-Val-Arg-NH-Mec substrate (Figure 3B). However, the proenzyme lacks this hydrolytic activity (data not shown).

**Table 1:** Michaelis-Menten constants (*K_m_*) and catalytic rate constants (*k_cat_*) for purified AcCP2 with Bz-Phe-Val-Arg-NH-Mec and Z-Arg-Arg-NH-Mec substrates. Data represent the mean values of three replicates ± SE (*n* = 3).

Proteinase	Substrates	*K*_m_ (μM)	*k*_cat_ (S^−1^)	*k*_cat_/*K*_m_ (Mm^−1^·S^−1^)
AcCP2 ^a^	Z-Arg-Arg-NH-Mec	72.3 ± 6.4	0.004 ± 0.001	0.005
	Bz-Phe-Val-Arg-NH-Mec	3.2 ± 1.1	15.4 ± 4.8	4812.5
Commercial bromelain ^b^	Z-Arg-Arg-NH-Mec	82.5 ± 10.2	0.003 ± 0.001	0.003
	Bz-Phe-Val-Arg-NH-Mec	4.6 ± 1.0	14.12 ± 5.2	3065

^a^ His-AcCP2 recombinant protein purified from yeast and incubated with pH 4.3 buffer; ^b^ Commercial bromelain preparations obtained from fresh pineapple fruit predominantly.

### 2.6. Disease Resistance Analysis in Transgenic Arabidopsis Plants

**Figure 4:**
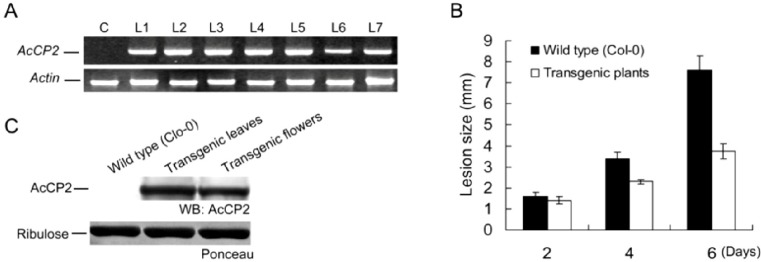
Disease resistance to fungal pathogen in transgenic *Arabidopsis* plants overexpressing *AcCP2*. (**A**) Detection of transgenic *Arabidopsis* plants using RT-PCR. *Actin* was used as an internal control; (**B**) Disease degree was showed on the leaves of wild-type (Col-0) and transgenic *Arabidopsis* plants 2, 4 and 6 days after inoculation; and (**C**) Western blotting analysis for AcCP2 protein in transgenic *Arabidopsis* plants. Ribulose is used as a control.

In our study, *Arabidop*sis is used as a heterologous expression system to test whether overexpression of *AcCP2* is important to inhibit the infection of fungal pathogens. Seven transgenic T0 seedlings with kanamycin-resistant phenotype were grown until flowering for T1 seed set. RT-PCR analysis shows a corresponding band to *AcCP2* in all transgenic T1 seedlings, but not in the control (Figure 4A). *X^2^* test of T1 seeds indicates that most transgenic lines contain one or more copies of insert genes in their genomes (data not shown). These data, thus, demonstrate *AcCP2* is successfully expressed in these transgenic *Arabidopsis* plants. And then, *B. cinerea* is selected to infect the transgenic lines to detect the resistance to fungal pathogen. Interestingly, severe disease symptoms appear on wild-type plants 6 days after inoculation, whereas these transgenic lines show much weaker symptoms by evaluation of necrosis sizes (Figure 4B). Immune blot analysis reveals that transgenic plants express an expected 25 kDa protein, which cannot be detected in wild-type (Clo-0) plants (Figure 4C). Though the mechanism of resistance increase has not been understood, it is clear that the constitutive expression of *AcCP2* from pineapple fruits in *Arabidopsis* can enhance resistance to *B. cinerea*.

### 2.7. Discussion

In the present study, an *AcCP2* encoding CP had been isolated by screening cDNA library of pineapple fruit, which is the sub-family C1A of papain-like CPs with sequence similarities (Figure S1C). It is synthesized as inactive pre-proenzymes with a signal peptide and a multifunctional N-terminal proregion proved by the analysis of catalytic activity (Figure 3). The N-terminal region plays an important role not only as inhibitors of enzymatic activity but for the correct folding of newly synthesized protein [12]. Furthermore, it could protect the protein against denaturing effects in sudden changes of pH conditions [12,20]. By contrast, AcCP2 also contains ERFNIN and GxNxFxD sequences (Figure 1B) like other members of C1A sub-family, which is thought to inhibit protein activity and should be removed before converting to the enzymatically active form [27,29,30]. This was evidenced that mature enzyme can be produced by mediating pH value of reaction buffer and cleaving the pre-sequence of AcCP2 proenzyme (Figure 2A). However, some papain subfamilies lack or contain only part of this sequence, resulting in a shorter proregion such as in cathepsin B [14]. Based on the predicted three-dimensional structure of AcCP2 (Figure S1B), Cys26 known as nucleophile, could be activated by His158 in the active sites [31]. Besides Cys26 and His158, Gln20 could help to form the electrophilic center that stabilizes the tetrahedral intermediate, while Asn179 is thought to orientate the imidazolium ring of catalytic His158 [29,32]. A single amino (cathepsin L5/L69Y) acid substitution can substantially influence the architecture of S2 subsite of *Fasciola hepatica* cathepsin L proteinases [33]. Therefore, we speculate that the active site of Cys 26 is supplemented by Asn 179 hydrogen bonding to His 158.

In the papain family, CPs demonstrate different catalytic activities to various substrates, including endopeptidases (cathepsins B, H, L and C), aminopeptidase (cathepsins H and C) and carboxipeptedase activity (cathepsin C) [34]. Fruit bromelain has also shown different activity to various protein substrates following different optimum pH and temperatures, such as at pH 7.7 for casein (59 °C) and at 6.5 for azocasein (55 °C) [22]. We found that AcCP2 purified from yeast had a high proteolytic activity against Bz-Phe-Val-Arg-NH-Mec, not for Z-Arg-Arg-NH-Mec (Table 1). However, AcCP2 peptidase precursor expressed in yeast lacks the proteolytic activity to Bz-Phe-Val-Arg-NH-Mec. That is because most heterologous expression systems for CPs produce proteolytically inactive proenzymes, which are stable at slightly acidic to slightly alkaline pH values [28]. Interestingly, the propeptide region of AcCP2 can be removed autocatalytically *in vitro* after incubation into acetate buffer (pH 4.3). However, other exopeptidases require an additional proteinase for full activation such as cathepsins X and C [35]. *AcCP2* transcripts can be detected in different development stages of pineapple fruits, which are consistent with the microarray analysis of gene expression profiles during fruit ripening [36], especially in outer sarcocarp. Although the reason and mechanism are not clear, plant proteinases are responsible for mediating a fundamental network of protein metabolism pathways during a life cycle [26,37]. In pineapple fruits, to keep the balance of sucrose-sink during fruit growth and development, invertases catalyze the irreversible hydrolysis of sucrose to glucose and fructose. From 40 to 80 days after anthesis, the ratio of hexose and sucrose is gradually decreasing with increase of the transcription levels of *AcCP2* (Figure S2A–B). However, the activity of invertase located in the vacuole is dramatically regulated to a negligible level (Figure S2C), especially in the core and basal sections [38]. In tomato, a CP has a dual function to regulate the gene expression of 1-aminocyclopropane-1-carboxylic acid synthase, which can affect the production of ethylene [39]. AcCPs synthesized in the ER as inactive precursors can be converted into the active forms by self-catalytic activity under acidic conditions, which implies that AcCPs maturation could take place in the vacuole. Therefore, these data together suggest the role of AsCP2 might regulate the level of acid invertase, or participate in alternating the cell wall texture during softening through protein metabolism based on the consistent tendency between *AsCP2* transcript characteristics and invertase abundance. Yamada *et al.* also found that CP was responsible for the maturity and activation of vacuole hydrolytic enzymes, which were involved in the degradation of cellular components sequestered from the cytosol by autophagy [40]. Plant CPs also participate in response to adverse environmental stresses, and possess remarkable toxicity against pathogens [4,41,42,43]. The classic examples demonstrated that papain or papain-like enzymes in the latex of papaya and fig (*Ficus virgata*) inhibited the growth of *lepidopteran larvae* [41]. We also found that overexpression of *AcCP2* in transgenic *Arabidopsis* plants improved disease resistance to *B. cinerea* (Figure 5B). Similar defense responses to pests and pathogens had been observed in other plants expressing *CP* genes [2,4,42,44,45]. Expression levels of AcCYS1 (*A. comusus* cystatin 1, an endogenous inhibitor of bromelain) are directly related with the resistance to blackheart in pineapple fruits, which implies that CP takes part in the defense response [43]. Increase of CP activity can enhance the degradation of gelatin-based jellies *in vitro* or the processing of a class IV chitinase in planta [46]. Whatever the mechanism is, our results indicate that *AcCP2* can be utilized for protecting plants from attack by fungal pathogens. In the future, further investigation is required to elucidate the interaction between CP and other proteins involved in the defense system.

## 3. Experimental

### 3.1. Plant Materials

Pineapple fruits (*Ananas comosus* cv. Comte de Paris) were selected from an orchard of the South Subtropical Crop Research Institute (SSCRI), Zhanjiang, Guangdong, China. The experimental materials used in this study were under the same management conditions such as irrigation, fertilization, soil management, disease control and pruning. Fifteen uniform fruits were randomly sampled every ten days throughout fruit development periods from the 20th day after anthesis between May and July in 2009. These fruits were immediately frozen in liquid nitrogen after sampling and stored at −80 °C. The sliced fleshes of five fruits were pooled together as one of three replications at each harvesting time. Seeds of *Arabidopsis thaliana* ecotype Columbia were surface-sterilized and allowed to germinate on agar plates (0.8%) [47]. The seedlings were transferred to soil 6 days after germination, and placed in a growth chamber at 22 °C under a 12 h light–dark cycle.

### 3.2. Isolation of AcCP2 Gene

By screening the cDNA libraries of pineapple fruits, a fragment with a polyA tail and 3'-untranslated region (UTR) showed high homology to CP. The 5'-full RACE kit (Takara, Dalian, China) was used to obtain its potential protein-coding region. Total RNA was extracted from pineapple fruits using Trizol Reagent (Invitrogen, Carlsbed, CA, USA). Poly(A)^+^ mRNA was isolated using the mRNA isolate kit (Omega, Norcross, GA, USA), and was transcribed into a single stranded cDNA according to the instruction of cDNA synthesis kit (Takara). Following the manufacturer’s protocol of 5' RACE kit (Clontech, Mountain View, CA, USA), 2 μL of cDNA was used as a template to amplify the 5'-terminal sequence using nested PCR primers (GSP1 and GSP2 for 5' RACE, Table S1). Finally, the PCR production was cloned into the pGEM-T Easy vector (Promega, Madison, WI, USA) for sequencing.

### 3.3. Extraction and Purification of Plant Proteins

Fruit proteinases were extracted from fresh pineapple (immature and mature) as described by Neuteboom *et al.* [27]. The major component proteinases within bromelain were separated by ammonium sulfate precipitation, dialysis and cationic exchange chromatography [19]. The bicinchoninic acid assay (Pierce Chemical, Rockford, IL, USA) was used to measure protein concentration.

*Arabidopsis* tissues were collected with forceps in 1.5 mL Eppendorf tubes containing 100 μL 2 × buffer [SDS/sample buffer, 125 mM Tris–HCl pH 6.8, 4% (w/v) SDS, 20% (v/v) glycerol, 2% (v/v) β-mercaptoethanol, 0.001% (w/v) bromophenol blue]. Plant materials were ground using a drill with a pestle-like bit in the Eppendorf tube until the mixture was homogeneous and immediately transferred to ice. All the extracts were resolubilized in the SDS buffer under the room temperature (20 °C), and were centrifuged at maximum speed (10 min, 13,000 g) to save the supernatant.

### 3.4. RNA Blot Analysis

Temporal and spatial expressions of *AcCP2* gene in pineapple fruits were measured using Northern blotting. Specific probes were produced by PCR amplification (Table S1, *AcCP*-F1b and *AcCP*-R1b) according to the manufacturer’s instruction of a DIG-labeled cDNA probe kit (Roche Diagnostics, Mannheim, Germany). *Actin* (HQ148720) probes were added as an internal control. The signals were detected using CDP-StarTM detection reagent (Amersham Biosciences, Bucks, UK) and following exposure to X-ray film.

### 3.5. Expression and Purification of pPIC9K-AcCP2 in P. pastoris

To obtain the expression production of recombinants in *P. pastoris*, cDNA coding the *AcCP2* protein was amplified using CP-F2c and CP-R2c primers (Table S1). The PCR production was digested with *Not* I as well as *EcoR* I, and then was ligated into pPIC9K vector. The recombinants were transformed into competent yeast cells (in *P. pastoris* strain GS115) and selection of Mut^+^ transformants was performed according to the *Pichia* Expression System Manual (Invitrogen, California, CA, USA). Recombinant plasmids of candidate clones were extracted by the Yeast Plasmid kit (Omega), and were tested by plasmid PCR using above primes. 300 μL of nickel-nitrilotriacetic acid (Ni-NTA) resin beads (Qiagen, Hilden, Germany) were mixed with the extracted proteins and gently stirred on a shaker at 150 rpm for 30 min. The mixture was washed with 3 volume of buffer (100 mM NaH_2_PO_4_, 10 mM Tris/HCl pH 6.8, 8 M Urea, pH 6.3). Finally, proteins were eluted with 1 mL of elution buffer (100 mM NaH_2_PO_4_, 10 mM Tris/HCl pH 6.8, 8 M Urea pH 4.5).

### 3.6. Antibodies and Western Blots

The constructed plasmids carrying pET30-*AcCP2* were expressed in *Escherichia coli* BL21 (DE3) cells. After incubation with 0.4 mM isopropyl-*β*-d-thiogalactopyranoside at 18 °C for 24 h, His-tagged AcCP2 protein was purified by nickel affinity chromatography according to the manufacturer’s protocol (Qiagen, Valencia, CA, USA). The protease factor Xa was used to remove the His tag from purified AcCP2 based on the supplier’s recommendations (New England Biolabs, Ipswich, MA, USA). The isolated protein was used for immunization of a New Zealand rabbit. For immune detection, recombinant proteins expressed in yeast were resolved by SDS-PAGE (12% w/v) and transferred onto a nitrocellulose membrane (Schleicher & Schuell BioScience, Dassel, Germany) by electroblotting (200 mA for 2 h, at 4 °C). Gels were stained either with Coomassie Brilliant Blue R-250 [48]. The membrane was subjected to immunodetection using secondary antibodies conjugated with a horseradish peroxidase (HRP)-conjugated goat anti-rabbit IgG antiserum (Boster, Wuhan, China). Immune blots were developed with a 3,3'-diamino-benzidine (Boster) as the chromogen.

### 3.7. Enzymatic Activity of Recombinant Proteinase

The proenzyme autocatalytic activation was assayed by incubating the purified proteins with various buffers after 1 h at 37 °C [9]. Catalytic activity was measured using the synthetic peptide substrates, Z-Arg-Arg-NH-Mec and Bz-Phe-Val-Arg-NH-Mec purchased from Sigma-Aldrich (St Louis, MO, USA), at 250 μg·mL^−1^ final concentration [20]. Peptidyl-NH-Mec substrate cleaved by proteinases can be detected colorimetrically at 410 nm in reaction buffer (10% dimethylformamide, 5 mM cysteine, 5 mM EDTA, and 0.1 M HEPES pH 7.3). Absorbance was measured at 30 s intervals for 20 min using a spectrophotometer (Shimadzu, Kyoto, Japan). The equivalent protein concentrations measured as above description were calculated from standards of known concentration in each assay.

The effect of pH on the enzymatic activity of the recombinant AsCP2 protein was investigated on 1% Bz-Phe-Val-Arg-NH-Mec substrate solution containing 15 mM CPs within the pH range 4.0–10 using citrate phosphate buffer (pH 3.0–7.0), Tris-HCl buffer (pH 8.0–9.0) and glycine-NaOH buffer (pH 8.0–11.0) with a constant temperature of 37 °C [49].

### 3.8. Measurement of Sugar Contents and Invertase Activity in Pineapple Fruits

The contents of glucose, fructose and sucrose were analyzed by high-performance liquid chromatography (HPLC, Shimadzu LC-6A; Kyoto, Japan), which was equipped with a RI detector and a SP1010 column (Showa Denko KK, Tokyo, Japan). The mobile phase was acetonitrile/water (75%/25%) at a flow rate of 0.5 mL·min^−1^. Invertase activity was extracted and assayed as described previously [38]. All assays were performed in triplicate and 30 fruits were used for each replicate.

### 3.9. Agrobacterium-Mediated Floral Dip Transformation of Arabidopsis

The full-length *AcCP2* cDNA in recombinant pGEM-T easy vector was amplified with the following AcCP-F2c and AcCP-R2c primers (Table S1) containing two introduced *BamH* I and *Xba* I restriction enzyme sites, respectively. The fragment was cloned into pCAMBIA1305 containing 35S promoter from pRT104 vector digested with the same enzyme digestion. pCAMBIA1305:35S:*AcCP2* recombinant plasmids were transformed into *Agrobacterium tumefaciens* (strain EHA105) competent cells via electroporation (1800 V) according to the protocol supplied by the manufacturer (Bio-Rad). Transformation was accomplished by simply dipping *Arabidopsis* inflorescences by *Agrobacterium* cells carrying the recombinants [47]. About 30 ng of genomic DNAs extracted using CTAB method [50] were used for PCR amplification with the primers (Table S1) in order to confirm the putative transgenic *Arabidopsis* plants.

### 3.10. Treatment of Arabidopsis Plants

For the whole-plant infection, three to four fully expanded leaves per plant were inoculated by spraying with a spore suspension of *B. cinerea* (5 × 10^5^ conidiospores ml^−1^ in 24 g L^−1^ potato dextrose broth, Difco, Detroit, ML, USA). Inoculated plants were placed in a growth chamber at 22 °C under a 12 h light–dark cycle. Relative humidity was maintained at 100% by covering the plants with clear plastic. Control plants were treated with only potato dextrose broth. Harvested leaves were photographed 2, 4, 6 days after inoculation and the diameters of lesion size were determined by Image J software in randomly selected areas of taken pictures. Data were acquired from four independent experiments (20 plants of per replicate) and 25 leaves were selected for each replicate. Analysis of variance was performed using statistix version 8.0 (Analytical Software, Tallahassee, FL, USA). Leave and flower samples from 10 transgenic plants were collected for AcCP2 protein detection, when were flowering.

### 3.11. Bioinformatic Analysis

Similarity search of the *AcCP2* sequence in public nucleotide or protein databases was performed using Blastn or Blastx algorithms [51]. The conserved domains were analyzed by online alignment [52]. A phylogenetic tree was constructed by the neighbor-joining (NJ) method using the NJ algorithm implemented in the Molecular Evolutionary Genetics Analysis (MEGA) software version 4.0.

For constructing three dimensional (3D) structure of AcCP2, a template for homology modeling was searched with BLAST program on Protein Data Bank [53,54]. Model was constructed in light of the root mean square deviation of C_α_ atoms between the modeled structure and the template structure within a reasonable range [9]. The consistency and viability of protein structure were evaluated and validated by PROCHECK software available online [55].

## 4. Conclusions

In our study, AcCP2 belonging to the CA1 subfamily was isolated from pineapple fruit. The functional domains were analyzed based on the molecular modeling and homology alignment. Transcripts of *AcCP2* gradually increased during fruit development until maturity. The pPIC9K-His-*AcCP2* recombinant was overexpressed in *P. pastoris*. The purified AcCP2 protein can be processed to a 25 kDa mature form after acid treatment (pH 4.3) *in vitro*. Optimum proteolytic specificity to Bz-Phe-Val-Arg-NH-Mec was showed at neutral. In additon, *AcCP2* expressed in *Arabidopsis* plants can also improve its resistance to fungal pathogens. This implies that *AcCP2* could take part in fruit development and resistance responses.

## Supplementary Materials

Supplementary materials can be accessed at: http://www.mdpi.com/10.3390/19/2/2374/s1.
