# Supplementary material for: Functional Properties of a Cysteine Proteinase from Pineapple Fruit with Improved Resistance to Fungal Pathogens in Arabidopsis thaliana
Source: Molecules. 2014 Feb 21;19(2):2374–89. doi: 10.3390/molecules19022374 (PMC6271751; doi:10.3390/molecules19022374)
Supplement: Supplementary file 1 [file molecules-19-02374-s001.pdf]

Supplementary Materials

Figure S1. Multiple alignment of AcCP2 with other plant CPs and presentation of 3D overall structure. (**A**) Comparison of the conserved amino acids located on N-terminal and C-terminal domains. Matching the complete consensus is shaded using blue color. The alignment was performed using Clustal W method in Megalign (DNAstar software). Proline and the motifs DWR, GAV, QG, CG, and CGSCW are almost present in all sequences; (**B**) 3D structure of AcCP2 demonstrated in cartoon style. Three disulfide bridges are represented with sticks. R-L domains and catalytic residues of AcCP2 are indicated with different colors and numbers. Columnar and lamellar characterizes are used to present α- helical and β-folding structures, respectively; and (**C**) Phylogenetic analysis of AcCP2 with other CPs (E.C.3.4.22 sub-sub-class) from different organisms. CPs with full prepropeptides are used to construct the unrooted tree by MEGA software version 4.0. Accession numbers and gene names from MEROPS proteinase are labeled in the evolution tree. This major CA::C1 cluster could be sub-divided in two groups according to amino acid sequences: Group I made up of the proteins more closely related to papain containing a mixture group, such as caricain, chymopapain, and cathepsins H, K and L; group II consisting of stem and fruit bromelain as well as ananain, which presents a weak internal branch.

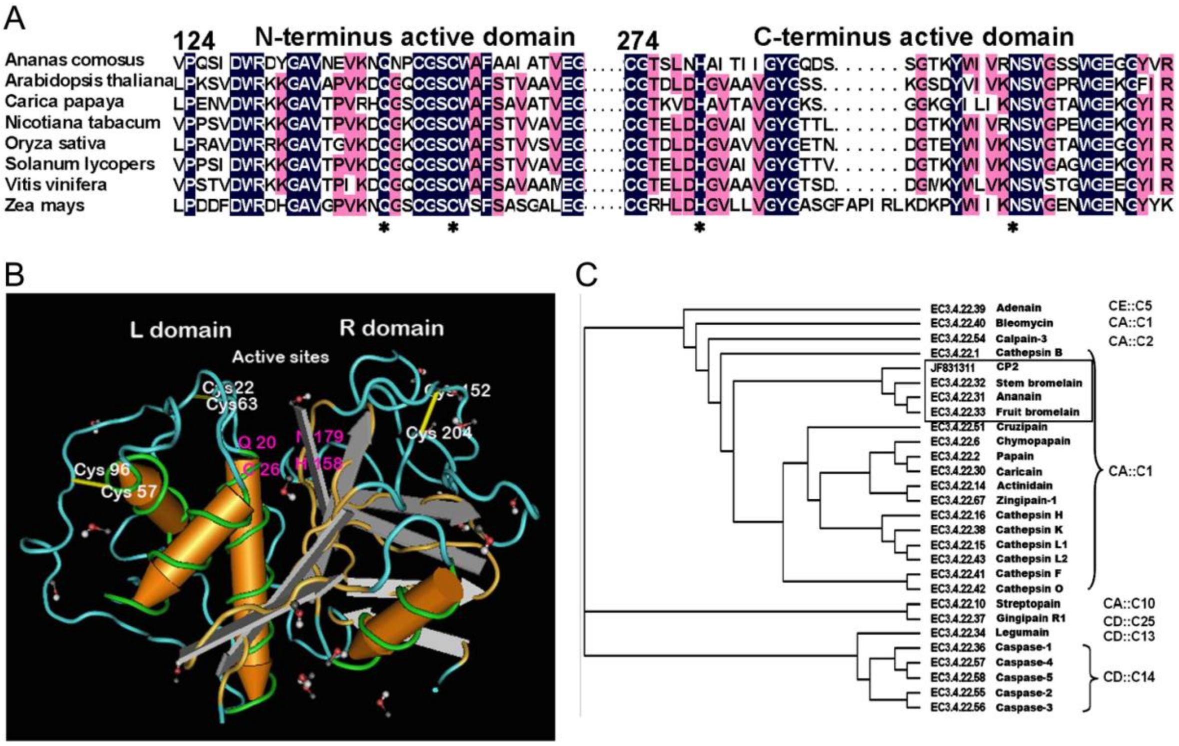

Figure S2. The changes of total sugar concentration (**A**), invertase (**B**) and the ratio of sucrose/hexose (**C**) during different development stages. Each point represent the mean values ± SE (n = 3).

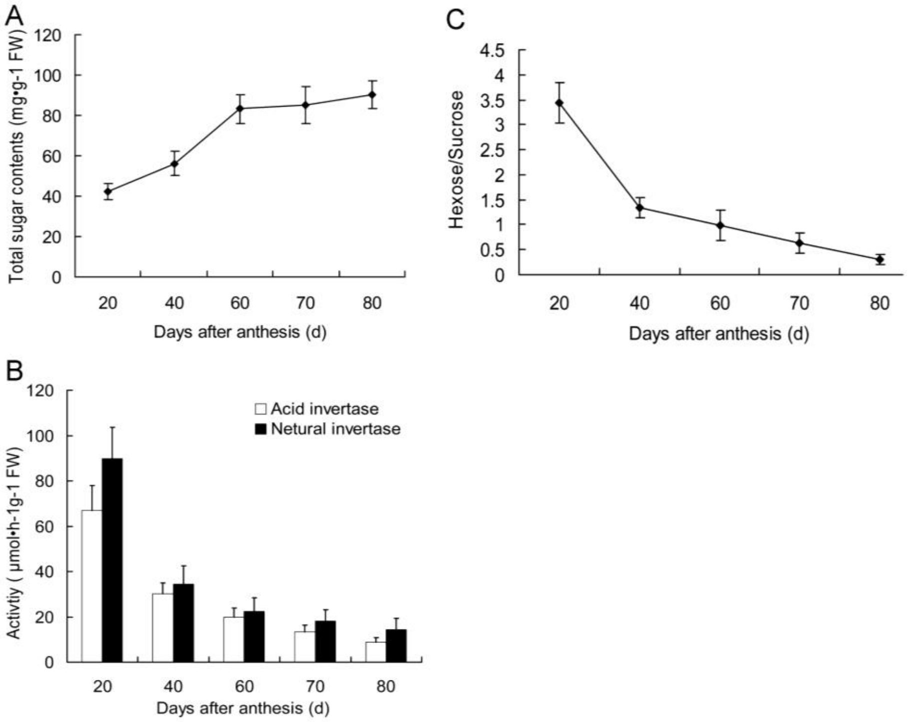

Table S1. Primer pairs used for PCR amplification in this study.

Primers	Sequences (5'-3')	Tm (℃)
GSP1 [a]	CCTGCCCGTAGCCTATAATGGTA	63
GSP2 [a]	CATCATGCTGCGTTCGTCGTTCC	63
AcCP-F1 [b]	GGTGCCGTAAACGAGGTCAAGAAT	62
AcCP-R1 [b]	TCCGCCCGTAGCCTATAATGGTAA	62
AcCP-F2 [c]	CGGGATCCCGGAACCCAGTGATCCCATGATG	65
AcCP-R2 [c]	GCTCTAGAGCCACCGAAACAGAACATAAACCACA	65
AtActin-F [d]	CAGTGTCTGGATCGGAGGAT	54
AtActin-R [d]	TGAACAATCGATGGACCTGA	54
AcActin-F [e]	GCGATGAGGCCCAGTCCAAGAG	65
AcActin-R [e]	TCACGGCCGGCAAGGTCCAGA	65

[a] Oligonucleotides for amplifying the full-length *AcCP2* gene; [b] Oligonucleotides for Northern blotting; [c] Oligonucleotides for constructing the expression vectors containing the *Not* I as well as *EcoR* I sites in forward and reverse primers underlined, respectively; [d] Oligonucleotides for *β-actin* sequence of *Arabidopsis* as internal control; [e] Oligonucleotides for *actin* sequence of pineapple fruit as internal control.
